# Xenotransplantation of Human glioblastoma in Zebrafish larvae: *in vivo* imaging and proliferation assessment

**DOI:** 10.1242/bio.043257

**Published:** 2019-05-15

**Authors:** Luis A. Vargas-Patron, Nathalie Agudelo-Dueñas, Jorge Madrid-Wolff, Juan A. Venegas, John M. González, Manu Forero-Shelton, Veronica Akle

**Affiliations:** 1 Laboratory of Neurosciences and Circadian Rhythms, School of Medicine, Universidad de los Andes, Bogota, 111711, Colombia; 2 Biomedical Sciences Laboratory, School of Medicine, Universidad de los Andes, Bogota, 111711, Colombia; 3 Biophysics Group, Department of Physics, Universidad de los Andes, Bogota, 111711, Colombia

**Keywords:** Glioblastoma, Zebrafish, Microscopy, Xenograft, Flow cytometry

## Abstract

Glioblastoma (GBM) is the most prevalent type of primary brain tumor. Treatment options include maximal surgical resection and drug-radiotherapy combination. However, patient prognosis remains very poor, prompting the search for new models for drug discovery and testing, especially those that allow assessment of *in vivo* responses to treatment. Zebrafish xenograft models have an enormous potential to study tumor behavior, proliferation and cellular interactions. Here, an *in vivo* imaging and proliferation assessment method of human GBM xenograft in zebrafish larvae is introduced. Zebrafish larvae microinjected with fluorescently labeled human GBM cells were screened daily using a stereomicroscope and imaged by light sheet fluorescence microscopy (LSFM); volumetric modeling and composite reconstructions were done in single individuals. Larvae containing tumors were enzymatically dissociated, and proliferation of cancer cells was measured using dye dilution by flow cytometry. GBM micro-tumors formed mainly in the zebrafish yolk sac and perivitelline space following injection in the yolk sac, with an engraftment rate of 73%. Daily image analysis suggested cellular division, as micro-tumors progressively grew with differentiated fluorescence intensity signals. Using dye dilution assay by flow cytometry, at least three GBM cells' division cycles were identified. The combination of LSFM and flow cytometry allows assessment of proliferation and tumor growth of human GBM inside zebrafish, making it a useful model to identify effective anti-proliferative agents in a preclinical setting.

## Abstract (precis)

**Summary:** The combination of light sheet fluorescent microscopy and flow cytometry allows assessment of proliferation and tumor growth of human GBM inside zebrafish, making it a useful model to identify effective anti-proliferative agents in a preclinical setting.

## INTRODUCTION

The incidence of primary malignant brain tumors is about five to six cases per 100,000 people per year, and glioblastoma (GBM) accounts for more than half of the cases, being the most common primary malignant brain tumor (Alifieris and Trafalis, 2015; Lapointe et al., 2018). GBM is also the deadliest form of brain tumor with a more than 90% mortality rate in a 5-year period (Alifieris and Trafalis, 2015). Currently, standard treatment consists of a multimodal approach including maximally possible surgical resection followed by a combination of radiotherapy and chemotherapy with temozolomide (Carlsson et al., 2014; Davis, 2016). Despite current advances, prognosis remains very poor with a median survival of about 15 months after diagnosis (Thakkar et al., 2014), and nearly 70% of patients have tumor recurrence within 9 months of initial treatment (Palti et al., 2012). The efficacy of current and new therapies have been limited by the heterogeneous biology of GBM cells, and the fact that they are located within the central nervous system (Chen et al., 2012). Therefore, new approaches for treatment discovery include the development of targeted drugs and personalized medicine (Holland and Ene, 2015), such as treatments based on evaluating *in vitro* sensitivity to drugs tested in primary GBM cell cultures from patients (Iwadate et al., 2003). However, *in vitro* models lack the microenvironmental signals, the interaction with extracellular matrix and the 3D structure in which tumors naturally grow (Katt et al., 2016). Consequently, there is a need to utilize new models for drug discovery and testing, especially those that allow reliable determination of *in vivo* responses to treatment, visualization of tumor microenvironment disruption and better correlation with clinical efficacy.

In recent years, new animal cancer models that are physiologically close to humans, are easier to manipulate and have the potential for high-throughput drug screening have been introduced in experimental research (Konantz et al., 2012). Zebrafish (*Danio rerio*), which stands as a promising *in vivo* model widely used in cancer research, display multiple advantages over their mammalian counterparts (Amatruda et al., 2002). Zebrafish reproduce easily, with hundreds of embryos obtained from a single mating that develop *ex utero* and can be manipulated at the embryonic stage. Also, the offspring are optically transparent in early life, which allows for the visualization of growing tumors with single cell resolution (Wyatt et al., 2017). Their small size, viability for incubation in multi-well plates and low cost provide the potential for high-throughput screening of antineoplastic drugs and up-scaling to increase the power of statistical analysis (Konantz et al., 2012). Human cancer can be injected into zebrafish by engraftment of cultured or patient-derived tumor cells (Vittori et al., 2015). Xenotransplantation of human cancer cells was introduced in 2005 by Lee et al. using melanoma cells (Lee et al., 2005). Since then, several models with a wide diversity of human tumors and xenograft protocols have been developed (Gansner et al., 2017; Idilli et al., 2017; Lee et al., 2005), including GBM (Geiger et al., 2008; Lal et al., 2011; Wehmas et al., 2016). Xenografts in zebrafish are usually performed during embryonic stages; hence, immunosuppression is not required since the adaptive immune system has not yet developed (Gansner et al., 2017; Lam et al., 2004). As opposed to mice, in which tumor growth takes from 15–21 weeks to be optimal for flow cytometry (O'Brien et al., 2007), tumor engraftment and growth is fast in zebrafish, providing readouts within a few days (Konantz et al., 2012; Vittori et al., 2015), making this model suitable for the evaluation of surrogate markers of clinical disease aggressiveness and response to treatment in the context of personalized medicine. Finally, antineoplastic drugs can be easily tested in zebrafish with xenografts since fish absorb small molecular weight compounds directly from water (Taylor and Zon, 2010).

Quantification of proliferation of human tumor xenografts in zebrafish embryos is of particular interest when assessing the response to treatment *in vivo*. Several approaches have been developed to assess proliferation of xenografted cells (Cabezas-Sainz et al., 2018; Corkery et al., 2011; Gamble et al., 2018; Haldi et al., 2006; Hamilton et al., 2016; Wehmas et al., 2016), but more automated and versatile assays are needed for their use in clinically relevant applications and drug discovery (Corkery et al., 2011).

The present study describes a robust assay to follow *in vivo* growth of tumors and quantify cell proliferation. The aim is to assess cell divisions and tumor growth by flow cytometry, but also to use light sheet fluorescence microscopy (LSFM) to follow tumors for long periods of time without phototoxicity. The combination of these techniques could be useful as a model to identify effective anti-proliferative agents against GBM and other solid tumors in a preclinical setting.

## RESULTS

### Injection of GBM cells does not affect zebrafish survival rates

**Fig. 1.**
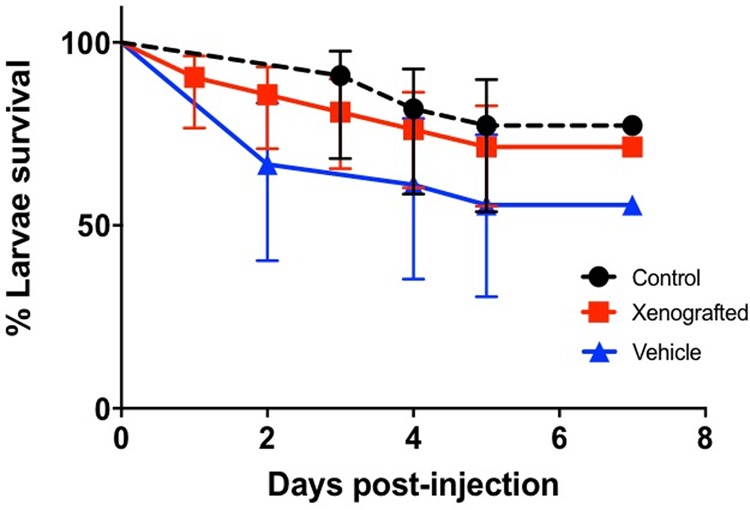
**Kaplan–Meier curve for zebrafish larvae survival after xenotransplantation.** Survival rate after 7 days post-injection was 80% for the microinjected larvae (*n*=30, vehicle or tumor cells) and 75% for the non-injected control group (*n*=8) maintained under the same conditions, including temperature (33°C). *P*=0.7, Mantel–Cox test.

In order to determine survival after microinjection, dechorionated zebrafish at 48 h post fertilization (hpf) were microinjected with either human GBM cells or vehicle (1× PBS) and maintained at 33°C. Daily examination under a stereomicroscope showed no difference in survival rates between tumor-cell-injected (71.4%, *P*=0.5, Mantel–Cox test) or vehicle-injected larvae (55.5%, *P*=0.1, Mantel–Cox test), both with respect to a control group (77.3%), which consisted of non-injected larvae maintained at the same conditions (Fig. 1). The engraftment rate was 73% on average, as determined by the number of micro-tumor-containing larvae at 24 h post injection (hpi) over the total number of microinjected larvae. The larvae that did not develop a tumor within the first 24 h after xenotransplantation remained tumor free.

### Human GBM micro-tumors persist up to 5 days post-injection

**Fig. 2.**
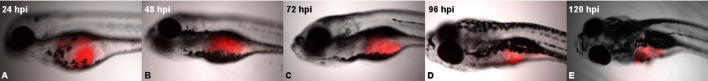
**Progression of a human GBM micro-tumor in a representative zebrafish larva by fluorescence stereomicroscopy.** Human GBM micro-tumors progress over time up to 5 days post-injection. Larva at (A) 24 hpi, (B) 48 hpi, (C) 72 hpi, (D) 96 hpi and (E) 120 hpi. 10X magnification.

After microinjection in the yolk sac, fluorescent micro-tumors formed in the yolk sac (Movie 1). Injected embryos tolerated the presence of human GBM cells for up to 5 days post injection (dpi), which was the last day of observation in our experiments. Engrafted micro-tumors visually increased in size, growing eccentrically from 24–72 hpi. Micro-tumors were detectable after this time point, but started regression (Fig. 2). Occasionally, labeled cells traveled through circulation and invaded distant sites throughout the larvae such as the tail, (Fig. S1). Some of those micro-tumors persisted with the main tumor, while others perished within 24 h.

### LSFM shows tumor proliferation and architecture

**Fig. 3.**
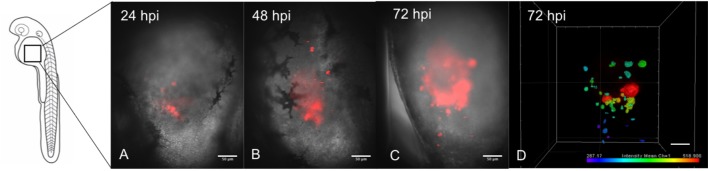
**Progression of a human GBM micro-tumor in a representative zebrafish larva by LSFM.** Larva at (A) 24 hpi, (B) 48 hpi and (C) 72 hpi. (D) Digital 3D reconstruction of the micro-tumor in C. Color scale indicates fluorescence intensity in each voxel (arbitrary units). Blue indicates low intensity and red indicates high intensity. Scale bars: 50 μm.

To have a more detailed assessment of GBM micro-tumors inside zebrafish larvae, LSFM was used. This technique provides high-resolution optical sections as in a confocal microscope, but with significantly reduced photodamage, making it possible to follow individual zebrafish over time with minimal phototoxicity. It was possible to locate the tumors with respect to anatomical structures of the fish by overlaying the fluorescence images with transmitted light images into a composite image. These images, as well as maximum intensity projection images of the fluorescence, showed an increase in tumor size from 24–72 hpi (Fig. 3A–C). While growing, new micro-tumor seeds appeared near engraftment sites, suggesting cell migration (Fig. 3B,C). Furthermore, 3D reconstruction of micro-tumors displayed differential fluorescence intensity signals with mass expansion. Cells emitting lower intensity were located in the periphery of the tumor mass. This dissipation of the tracer suggests tumor cell division inside the larvae (Fig. 3D)*.*

### Cell proliferation by flow cytometry

**Fig. 4.**
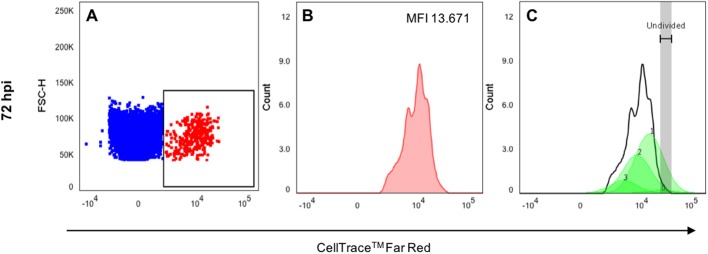
**Tumor cell proliferation by flow cytometry.** Several human GBM cell generations could be identified using a dye dilution assay by flow cytometry. Enzymatic dissociation of a tumor-containing zebrafish larva at 72 hpi. (A) Dot plot showing the relative size (FSC) in the *Y* axis and fluorescence in the *X* axis. Human astrocytes labeled with CellTrace™ Far Red fluorochrome showed in the square gate. (B) Histogram showing the fluorescent intensity on the gated human astrocyte population. (C) Proliferation modeling depicting human GBM cell proliferation up to four generations. Numbers on the peaks of green curves indicate the cell generation.

**Fig. 5.**
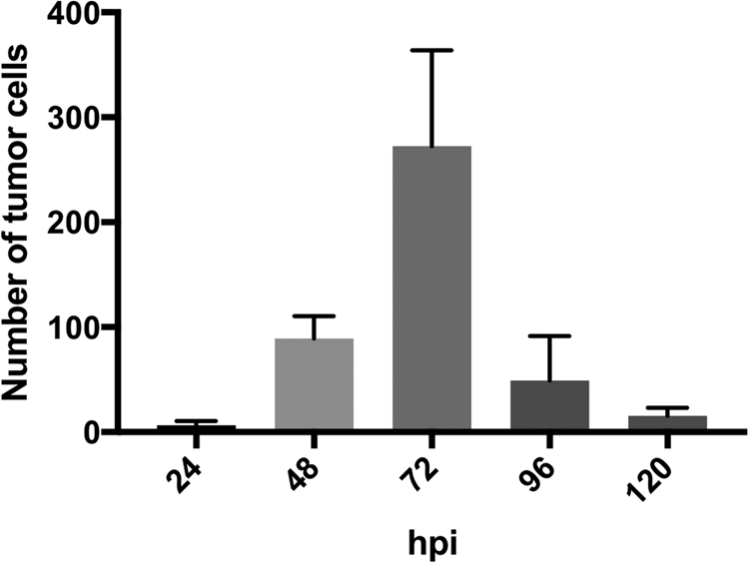
**Number of human GBM cells according to time post-injection assessed by flow cytometry.** Fluorescent cells detected after enzymatic digestion of larvae containing human GBM micro-tumors. Each larvae sample was run in a flow cytometer and cells gated as shown in Fig. 4A. The number of tumor cells detected decreased during the first 24 h, and then increased up to 72 hpi. Larvae in each time point *n*=5.

To confirm if human GBM cells proliferate in zebrafish, larvae containing micro-tumors were enzymatically digested at various time points post-injection to obtain cellular suspensions for flow cytometry analysis. The cellular complexity and size parameter profile obtained for fish and human GBM cells were similar. To discriminate between them, the fluorescence intensity signal of the CellTrace™ Far Red fluorochrome was assessed using the 660/20 nm detector, as shown in the dot plot in Fig. 4A. Gating of this population showed differential fluorescence intensity of the tumor cells, as shown in the histograms in Fig. 4B. These histograms were analyzed using the proliferation modeling tool of FlowJo^®^ software to determine the number of cell generations. Various GBM cell populations were identified based on differential fluorescence intensity, which represented four cell generations at 72 hpi including the original population that was injected. This data suggests at least one division cycle every 24 h for part of the tumor cells (Fig. 4C). The number of human GBM cells identified by flow cytometry from enzymatically digested zebrafish larvae increased up to 72 hpi with time after injection. After 72 hpi, this value started to decrease (Fig. 5).

## DISCUSSION

In the last few decades, zebrafish has emerged as an alternative animal model for cancer studies. Experiments with different human tumor cell lines have established zebrafish as a promising model for drug discovery and testing (Amatruda et al., 2002; Corkery et al., 2011; Fior et al., 2017; Geiger et al., 2008; Idilli et al., 2017; Konantz et al., 2012; Nicoli and Presta, 2007; Yang et al., 2013). Here, we successfully micro-injected labeled GBM cells in zebrafish larvae and followed micro-tumor progression using a combination of stereomicroscopy, LSFM and flow cytometry. These tools make it possible not only to follow the fluorescent xenograft and assess micro-tumor size, shape and brightness, but also to quantify discrete number of tumor cells and cellular divisions over time. To our knowledge, this is the first time that these complementary tools have been used simultaneously in this context, providing a good alternative to study tumor evolution over time *in vivo*.

A common concern with the xenograft model is the difference in incubation temperature between mammalian cells and zebrafish embryos, given that the optimal temperature for incubation is 28.5°C for the embryos (Kimmel et al., 1995). However, it has been reported that zebrafish embryos develop normally if they are kept between 25°C and 33°C (Kimmel et al., 1995), and that xeno-injected embryos kept at 34°C and 36°C have high survival rates of 95% and 87.5%, respectively (Cabezas-Sainz et al., 2018). Indeed, a variety of incubation temperatures for GBM xenografts in zebrafish have been proposed from 31°C (Vittori et al., 2016) to 33°C (Gamble et al., 2018; Wehmas et al., 2016). Even varying the incubation temperature between 28°C and 35°C had no effect on the survival of cells after engraftment (Geiger et al., 2008)*.* Here, we used the temperature of 33°C, since it does not affect embryo viability, and dye dilution assay by flow cytometry confirmed that the GBM cell line used here proliferates at this temperature*.* Also, there was an acceptable engraftment rate and embryo viability. It is worth noting that higher peaks denoting proliferation were detected when the tumors grow inside the fish, than *in vitro*, in the same time period, indicating that GBM cells are able to grow at 33°C. This in turn, suggests that the zebrafish microenvironment might be better suited to test and validate treatments that *in vitro* samples.

It has been hypothesized that injecting human cancer cells into the yolk sac may provide the advantage of less susceptibility to tissue microenvironment signaling and a better chance for the cells to retain their cancer phenotype (Lee et al., 2005), while at the same time providing a nutrient rich acellular compartment where cell proliferation is supported (Haldi et al., 2006). Thus, the site of injection can be helpful at reducing the effects of environmental cues and space limitations in cell proliferation, providing a suitable *in vivo* matrix, as observed in our experiments. Even though previous studies show that the brain might be a more permissive environment for GBM growth (Hamilton et al., 2016; Wehmas et al., 2016; Welker et al., 2016b), we chose to microinject human tumor cells in the yolk sac, which is a prominent structure at 48 hpf, to avoid spatial constrains for the tumor.

Previous attempts to quantify tumor cell proliferation in the context of zebrafish xenograft assays involved the enzymatic dissociation of tumor-containing larvae into a single-cell suspension and either manual enumeration of fluorescently labeled human cells using a hemocytometer (Haldi et al., 2006), or semi-automated counting on micrographs (Corkery et al., 2011). Other reports also appraise proliferation of xenografted cells by analysis of embryo demise (Pruvot et al., 2011). In the case of GBM xenografts, to estimate proliferation, several studies have used image analysis of intensity using fluorescence microscopy and automatically or manually delimited the fluorescent volume (Gamble et al., 2018; Geiger et al., 2008; Wehmas et al., 2016), obtaining a reliable assessment of tumor growth, especially for live animals over time. Recently, Pudelko et al. used Ki67 immunohistochemistry in cryosections of zebrafish embryos transplanted with GBM tumors to show tumor cell division (Pudelko et al., 2018). While this method provides an accurate assessment of proliferating cells, dilution-based proliferation assessment is a simpler quantitative approach, useful for both imaging and cytometry. The CellTrace™ Far Red Cell Proliferation dye has low cellular toxicity, binds to amine proteins inside the cell (Filby et al., 2015) and can be detected up to eight generations in human cells (Zhou et al., 2016). Additionally, using flow cytometry, we were able to accurately determine the total number of human cells in individual zebrafish larvae at different time points after injection, and model the number of cell divisions, reflecting the evolution of the tumor.

LSFM allowed us to follow the proliferation of tumors in individual fish for several days without compromising their viability. Compared to confocal or epifluorescence microscopy, LSFM is less phototoxic, allowing for imaging over longer periods of time. Previous works have used confocal (Gamble et al., 2018; Welker et al., 2016a) and epifluorescence (Geiger et al., 2008) imaging to track GBM in zebrafish individuals over time, imaging the progression of tumors at several time points. Recently, LSFM has been used to followed GBM cells in zebrafish to observe the tumor and determine its volume after treatment (Pudelko et al., 2017, 2018).

Here using LSFM, we tracked tumor growth of individual specimens daily for up to 5 days and for many hours at a time, produced 3D reconstructions of the tumor, and additionally digested individual embryos for flow cytometry analysis. LSFM permitted to evaluate changes in size and fluorescence intensity of cell clusters, which could be translated into estimates of tumor proliferation, with the limitation that this type of dye made it difficult to segment individual cells when in clusters. On the other hand, dye-dilution could make it possible to xenograft primary cells (Pudelko et al., 2018), for which fluorescent proteins and antibodies are of limited use *in vivo*, and could even be complemented with membrane dyeing for better segmentation.

To the best of our knowledge, we are the first to use LSFM in addition to flow cytometry for the assessment of tumor proliferation in live zebrafish. This combination of methods enables rapid and reliable quantification, capable of detecting small changes in tumor cell number under different conditions and for several consecutive days. In particular, both LSFM and flow cytometry could be compatible with high-throughput screening, making the assessment of pharmacological agents at different concentrations and time points efficient. Using these tools will aid in the assessment of the effect of specific drugs in distinct GBM tumors, making the evaluation of adequate treatment for a particular patient a realistic possibility.

## MATERIAL AND METHODS

### Astrocytoma cell culture

Human astrocytoma cell line (ATCC^®^ CRL-1718™) obtained from the American Type Culture Collection (Manassas, VA, USA) was cultured in T25 culture flasks (Corning, NY, USA) at a density of 2×10^5^ cells approximately in 4 ml of RPMI-1640 medium (Sigma-Aldrich, St. Louis, MI, USA) with 10% of fetal calf serum (FCS) (Eurobio, Les Ulis, France), supplemented with 2 mM L-glutamine, 4.5 g/l glucose, 10 mM HEPES, 1 mM pyruvate, and 1% penicillin-streptomycin (all from Gibco, Grand Island, NY, USA). Cultures were maintained at 37°C and 5% CO_2_ environment. Once the monolayer was confluent, cells were detached using 2 ml of 0.25% trypsin-EDTA 1X (Gibco) and incubated for 3 min at 37°C. Cells were visually checked for detachment. Trypsin solution was blocked using 4 ml of RPMI-1640 with 10% FCS in a 15 ml tube. Cell suspension was centrifuged for 5 min at 1350×***g*** (Sorvall, Thermo Fisher Scientific, Waltham, MA, USA). Cells were manually counted in a Neubauer chamber and checked for viability using Trypan Blue (Sigma-Aldrich) in a light microscope at 40X magnification. Cellular pellet was re-suspended in 4 ml of complete media and used to create new cell cultures using 2×10^5^ cells per T25 culture flask.

### Fluorescent cell staining

Detached 2×10^6^ cells were washed and centrifuged in phosphate buffer solution 0.01 M pH 7.4 (PBS 1×). Cells were re-suspended with CellTrace™ Far Red (Invitrogen, Thermo Fisher Scientific) solution 1:1000 dilution in PBS 1X and incubated at 37°C bath Marie for 20 min, mixing gently every 5 min. Then, 4 ml of PBS 1X with 10% of FCS was added and incubated for 5 min at 37°C. All solutions were pre-heated at 37°C before use. After incubation, cells were centrifuged for 5 min at 1350×***g***, the supernatant discarded and cells re-suspended in sterile PBS 1× at a concentration of at least 1×10^6^ cells per ml. To ensure low residual volume in the cellular pellet, the cell suspension was transferred to 1.5 ml vials and centrifuged for 5 min at 3250×***g***. The supernatant was discarded, the vials were placed upside down for a minute and cells re-suspended for a final concentration of approximately 100 cells/nl. At least 10,000 cells were collected and re-suspended in FACs Flow solution (BD Biosciences, San Jose, CA, USA) for staining control, read at 660/20 nm in a FACsCanto II cytometer (BD Biosciences) and analyzed using FacsDiva 6.1 software (BD Biosciences).

### Animal care and maintenance

Adult zebrafish (*Danio rerio*) TAB wild type were housed and reared on a 14 h light-10 h dark cycle, at 28°C, according to standard protocols (Westerfield, 2000) in a controlled multi-tank recirculating system (Aquaneering, CA, USA). Embryos were collected by natural spawning and raised in egg water (60 µg/ml sea salt in RO water with 1 ppm Methylene Blue) at 28.5°C until 48 hpf. Staging was performed as previously reported (Kimmel et al., 1995). After injection of human GBM cells, larvae were maintained at 33°C. All protocols were approved by the Institutional Animal Care and Use Committee of Universidad de los Andes (CICUAL) through the protocol FUA 18-007 from 2018.

### Zebrafish larvae cell micro-injection

Needles were prepared from borosilicate glass capillaries of 0.75 mm internal diameter without filament (World Precision Instruments, FL, USA) using a micropipette puller (Narishige, Tokyo, Japan). Needles were cut using a scalp under the stereoscope, creating a blunt open to obtain 1–3 nl per injection. Zebrafish of 48 hpf were removed from the chorion, anesthetized with 200 mg/l Tricaine (Sigma-Aldrich) and mounted in agarose 0.1% gel bed. The CellTrace™ Far Red labeled GBM cells were loaded into the glass needle at a density of about 3000cells/ml. Each anesthetized larva was microinjected about 100 GBM cells into the duct of Cuvier at 1.5–3 psi, using a micro-injector (Narishige). After injection, larvae were transferred to fresh egg water for recovery during 1 h at 28±0.5°C, and then incubated at 33°C for the rest of the experiment.

### Enzymatic larvae digestion and flow cytometry analysis

Larvae with micro-tumors detected by fluorescence microscopy were enzymatically digested at different hpi to be analyzed by flow cytometry. Larvae were first anesthetized with 0.004% tricaine, transferred to 1.5 ml vials with calcium-free Ringer's solution 116 mM NaCl, 2.9 mM KCl, 5 mM HEPES, pH 7.2 (all reagents from Sigma-Aldrich) and incubated at room temperature for 15 min. Larvae were transferred to a 35 mm culture dish (Falcon, Franking Lake, NJ, USA) with 2 ml of trypsin 0.25% EDTA 1x solution (Gibco), incubated at 37°C and dissociation was mechanically assisted by performing up and down pipetting with a 200 µl tip every 3–4 min until a single cell suspension was visualized under the microscope (∼15 min). Trypsin was neutralized using twice the volume of 5% FCS in PBS 1×. Cell suspension was centrifuged at 3250×***g*** for 10 min. Cell pellet was re-suspended in 100 µl of FACSFlow and run on a FACS canto II cytometer. Human cells were detected at 660/20 nm with excitation of the He-Ne 630 nm laser, to be differentiated from larvae cells. Fluorescent positive cell population was gated to carry out the proliferation analysis with FlowJo^®^ 10.3 software (Tree Star, Inc. Ashland, Oregon, USA) to estimate the number of cell generations.

### LSFM

A custom-made LSFM was used to image the larvae. In the optical setup, the beam from a 633 nm diode laser (06-MLD, Cobolt AB, Solna, Sweden) was expanded by means of a Galilean telescope composed of a pair of f=30 mm and f=200 mm achromats (Edmund Optics, Barrington NJ, USA). An achromat cylindrical lens of f=70 mm (Thorlabs, Newton, NJ, USA) focused the expanded beam into a line at the back pupil of an N Plan 10× NA0.3 air objective (Leica, Wetzlar, Germany). This microscope objective was used for illuminating the sample, which was placed within a chamber. The chamber was filled with egg water kept at 33°C by means of a custom temperature controller. A water-immersion physiology CFI Plan Fluorite 40× NA0.8 (Nikon, Tokyo, Japan) collected light from the specimen, which we filtered with a bandpass filter (ET700/75, Chroma, VT, USA). An f=200 achromat (Edmund Optics) projected the image on an sCMOS camera (Neo 4.5, Andor, Belfast, Ireland). To produce tomographic views of the fish larvae, the light sheet was maintained at a fixed position while the sample was displaced at 1 μm steps using a micromanipulator (Sutter Instruments, Novato, CA, USA). An Acousto Optical Tunable Filter (AOTFnC-400.650, AAOptoElectroncis, Orsay, France) placed before the Galilean telescope was used to further modulate the intensity so that no more than 1.5 mW reached the back pupil of the illumination objective and to block the light during sample displacement. The exposure time for each frame was set at 200 ms, with the sensor cooled to −20°C. The instrument was controlled with an in-house LabView application (National Instruments, Austin, TX, USA), allowing for automated acquisition of *z*-stacks and time-lapses.

### Image processing and visualization

Image processing and visualization were performed using FIJI ImageJ (Schindelin et al., 2012) and Bitplane Imaris 8.2.0 software (Oxford Instruments, Zurich, Switzerland). Images from the stereomicroscope and LSFM were visualized first in FIJI ImageJ, and brightness and contrast levels of fluorescence images were adjusted at the same levels for comparison. Transmitted light (gray channel) and fluorescence images (red channel) were overlaid to provide anatomical context of tumor progression over time. For LSFM images, maximum intensity projections (MIP) for planes of interest (red channel) were overlaid with a transmitted light image. In Bitplane Imaris, 3D visualizations of the datasets as volumes in the 3D view were obtained, and the intensity range of the fluorescence channel and the gamma correction was changed to improve image display. Then, surface reconstructions of the tumors were created, which allowed applying a color-coded intensity scale to visualize the spatial distribution of micro-tumors, with the highest and lowest fluorescence intensity as a surrogate indicative of cell proliferation.

### Data analysis and statistics

GraphPad Prism software version 7.0 (GraphPad Software, San Diego, CA, USA) was used to build graphs and diagrams and to perform Mantel–Cox statistical test for comparison of survival curves and calculation of engraftment rate. Data is presented as means with standard error bars.

## Supplementary Material

Supplementary information
