# Supplementary material for: Xenotransplantation of Human glioblastoma in Zebrafish larvae: in vivo imaging and proliferation assessment
Source: Biol Open. 2019 May 15;8(5):bio043257. doi: 10.1242/bio.043257 (PMC6550087; doi:10.1242/bio.043257)
Supplement: Supplementary information [file biolopen-8-043257-s1.pdf]

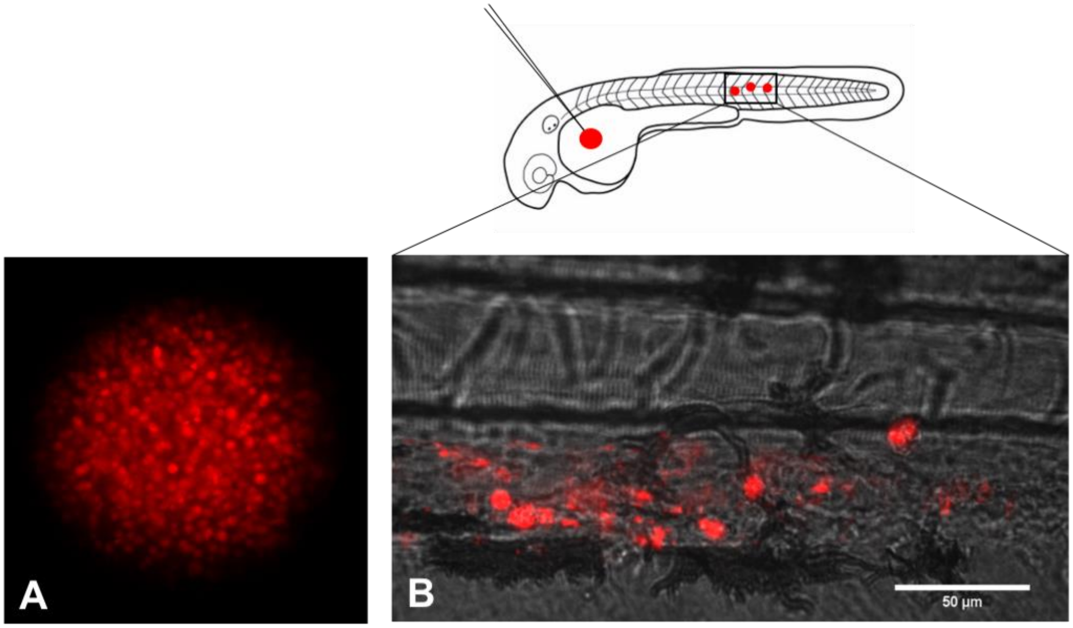

Figure S1. Injection of fluorescent human GMB cells into zebrafish larvae. Larva drawn to depict the injection site (tip) at the yolk sac and a location of where cells migrate to (square). A) Calibration drop containing fluorescent cells for injection using the microinjector. B) Florescent tumor cells traveling in the circulatory system.

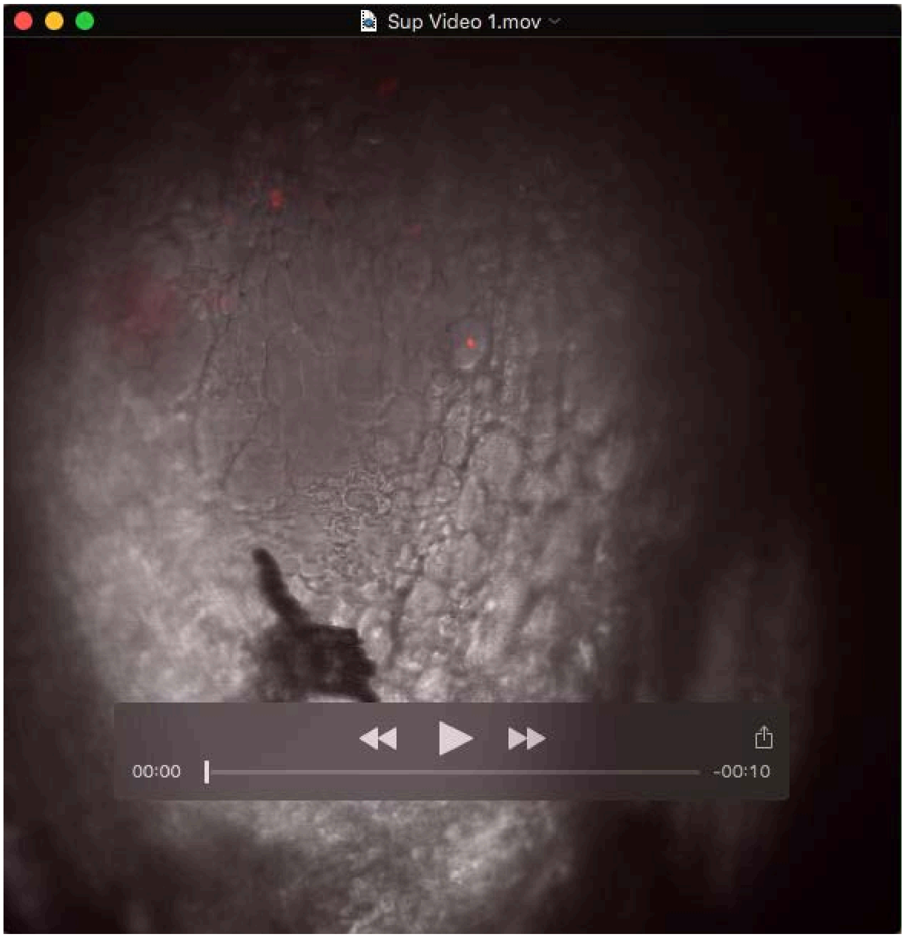

Movie 1.

Biology Open • Supplementary information
